# Impact of increasing levels of adaptive statistical iterative reconstruction on image quality in oil-based postmortem CT angiography in coronary arteries

**DOI:** 10.1007/s00414-021-02530-1

**Published:** 2021-02-24

**Authors:** Andrea Steuwe, Judith Boeven, Lena Cordes, Stefano Draisci, Johannes Boos, Silke Grabherr, Christine Bruguier, Hannan Dalyanoglu, Gerald Antoch, Joel Aissa

**Affiliations:** 1 Medical Faculty, Department of Diagnostic and Interventional Radiology, University Dusseldorf, D-40225 Dusseldorf, Germany; 2 University Center of Legal Medicine Lausanne-Geneva, University Hospital of Lausanne, Chemin de la Vulliette 4, 1000 Lausanne 25, Switzerland; 3 Department of Cardiovascular Surgery, Heinrich-Heine University Hospital, D-40225 Dusseldorf, Germany

**Keywords:** Computed tomography (CT), Postmortem CT coronary angiography, Iterative reconstruction, Image quality, Forensics

## Abstract

**Introduction:**

Postmortem multi-detector computed tomography (PMCT) has become an important part in forensic imaging. Modern reconstruction techniques such as iterative reconstruction (IR) are frequently used in postmortem CT angiography (PMCTA). The image quality of PMCTA depends on the strength of IR. For this purpose, we aimed to investigate the impact of different advanced IR levels on the objective and subjective PMCTA image quality.

**Material and methods:**

We retrospectively analyzed the coronary arteries of 27 human cadavers undergoing whole-body postmortem CT angiography between July 2017 and March 2018 in a single center. Iterative reconstructions of the coronary arteries were processed in five different level settings (0%; 30%; 50%; 70%; 100%) by using an adaptive statistical IR method. We evaluated the objective (contrast-to-noise ratio (CNR)) and subjective image quality in several anatomical locations.

**Results:**

Our results demonstrate that the increasing levels of an IR technique have relevant impact on the image quality in PMCTA scans in forensic postmortem examinations. Higher levels of IR have led to a significant reduction of image noise and therefore to a significant improvement of objective image quality (+ 70%). However, subjective image quality is inferior at higher levels of IR due to plasticized image appearance.

**Conclusion:**

Objective image quality in PMCTA progressively improves with increasing level of IR with the best CNR at the highest IR level. However, subjective image quality is best at low to medium levels of IR. To obtain a “classic” image appearance with optimal image quality, PMCTAs should be reconstructed at medium levels of IR.

## Introduction

Postmortem multi-detector computed tomography has become an important part in forensic imaging. The number of postmortem CT (PMCT) examinations has increased significantly over the last decades such that PMCT has established into forensic routine [1]. Studies indicate an important impact of PMCT on the detection rate of relevant findings compared to conventional autopsy [2, 3]. Moreover, the quality of diagnostic performance increases by combining these methods [3].

However, unenhanced PMCT, as opposed to contrast-enhanced CT, is limited by low soft tissue contrast, associated with the disability to detect important findings like aortic ruptures or myocardial infarction [1, 4, 5]. Resulting from this limitation, the contrast media-based postmortem CT angiography (PMCTA) has been introduced. According to Grabherr et al., PMCTA improves the detection of soft tissue and vascular findings compared to PMCT performed without contrast media and conventional autopsy [4, 5]. Therefore, PMCTA has become an important part of the workup routine in forensic imaging [5]. Current studies demonstrate that postmortem coronary CT angiography (PMCCTA) could be considered a valid and useful tool for the detection of coronary artery disease and myocardial infarction [6]. Since the introduction of PMCTA, different techniques and contrast substances, such as ioxithalamate, diesel oil, and oily liquids, were analyzed [7–9]. Nowadays, multiphase PMCTA protocol using oily contrast agents has established into forensic routine and reflects the worldwide most used angiographic technique [5]. In literature, a different coronary PMCTA technique called targeted coronary angiography (TCA) is introduced [10, 11]. TCA is a less invasive method which injects the contrast agent via a blocked urinary catheter into the ascending aorta and thereby in the coronary arteries [10, 11]. Studies analyzing the performance of TCA revealed a good correlation for identifying coronary lesions and therefore for investigating CAD, by comparing with conventional autopsy and histological investigations [12, 13].

Iterative reconstruction (IR) techniques have been introduced into radiological CT diagnostic as a reliable method to reduce image noise [14]. The underlying method of IR techniques varies with vendor and software. In general, IR methods use correction loops to progressively refine image data and to separate image information from noise. The reduced image noise can be used to lower the radiation exposure while maintaining image quality when imaging living patients [14]. IR techniques are nowadays frequently used in clinical routine as well as in postmortem CT and have replaced the traditional filtered back projection (FBP) in many applications. However, studies reported a limited value of higher IR levels due to altering of the “classic” image appearance, causing a blotchy or aquarelle-like image impression [15, 16]. Noise suppression may reduce the coarseness of images but might at the same time remove small details which impedes the diagnostic quality of an image, though recent advanced IR systems, such as model-based IR, are even more powerful with respect to reducing and preventing artifacts than adaptive statistical IR [16–19].

Since IR reduces image noise and while clinical radiologists are used to the image impression of IR-processed images in daily routine, it is desirable to acquire images in PMCT using the same reconstruction method. However, the optimal choice of the level of noise suppression depends on the type of CT examination. In PMCT, the situation is different from the clinical setting and dose reduction is less relevant. Because the higher radiation dose in PMCT results in less image noise compared to clinical routine CT, optimal IR levels for noise suppression may differ between PMCT and clinical CT examinations. Further, the IR level specifically needs to be evaluated for different PMCT protocols (e.g., PMCT of the coronary arteries) in terms of quantitative and qualitative image quality [20]. For this purpose, we aimed to investigate the impact of different levels of IR on the objective and subjective image quality in PMCTA.

## Materials and methods

### Cadavers

We retrospectively analyzed the coronary arteries of 38 human cadavers undergoing whole-body postmortem CT angiography and conventional autopsy between July 2017 and March 2018 in a single center. As this was a retrospective study and data were treated anonymously, a specific ethical approval was not necessary according to an agreement with the local justice department and the ethical standards. The cadavers showed different causes of death, e.g., myocardial infarction, intoxication, trauma, or death by drowning. All cadavers received a full-body PMCTA after a detailed forensic examination and prior to conventional autopsy. As the time of death is unknown, an exact calculation of the postmortem interval (death to PMCT) cannot be given; however, it ranged from half a day to 3 days maximum (with storage in the refrigerating room).

**Fig. 1:**
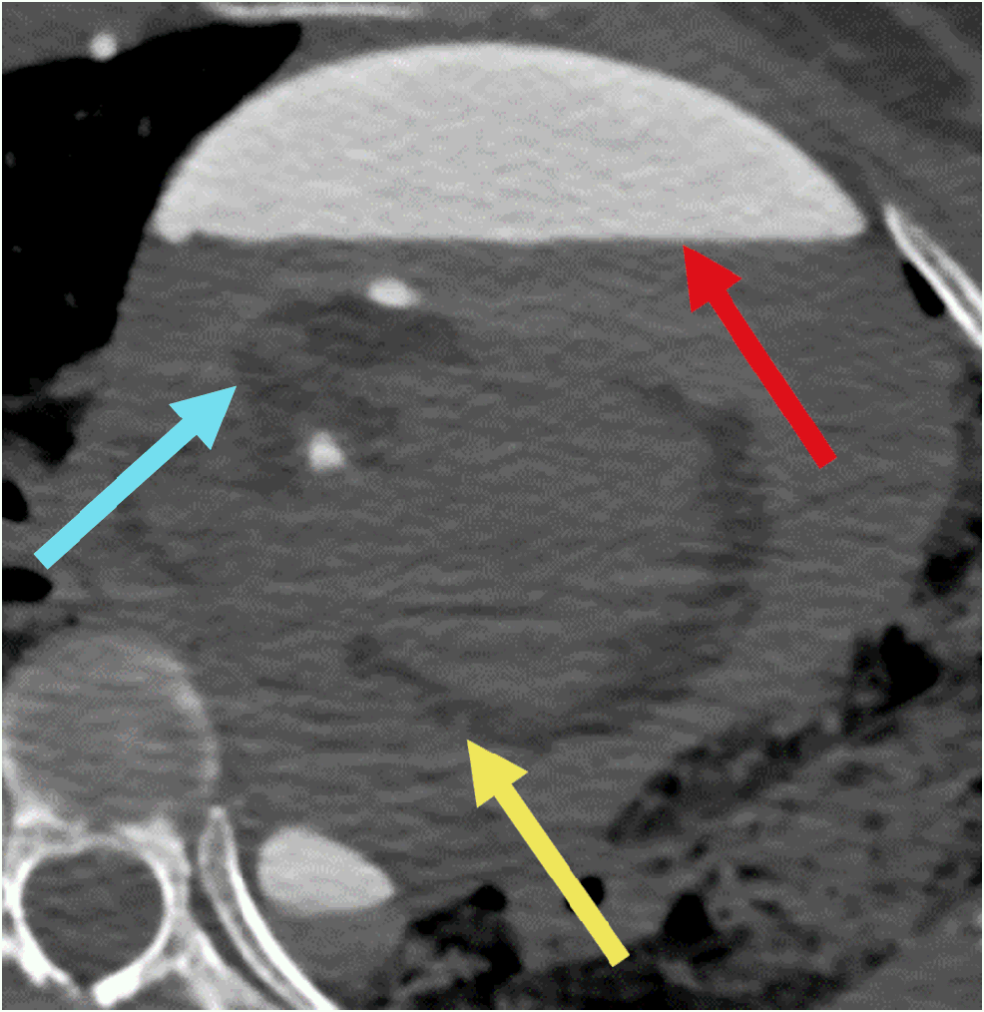
Failed contrast enhancement of an excluded cadaver due to post-traumatic coronary leakage. Red arrow points to pericardial hematoma with contrast extravasation. Blue arrow points to the non-enhanced coronary artery and the yellow arrow points to the non-enhanced left circumflex coronary

Of the 38 examined cadavers, 10 cadavers were eventually excluded from the analysis due to lack of contrast in the coronary arteries and 1 due to heavily calcified coronary arteries (see Fig. 1). The remaining 27 cadavers included 9 female and 18 male cadavers with a mean age of 55.5 ± 19.7 years (range 19–92 years).

### Scan protocol

Three-phasic (arterial, venous, and dynamic) PMCTA was performed on a 64-row CT scanner (GE LightSpeed VCT, GE HealthCare, Milwaukee, WI, USA) by using the standardized protocol with the following scan parameters: tube potential 120 kV_p_, automatically modulated tube current 200–400 mA, noise index 20, pitch 0.984, tube rotation time 0.8 s, and scan field of view (FOV) 50 cm (see Table 1). Tube current was automatically modulated to obtain a constant image quality within a cadaver. The CT acquisition covered the vertex to the trochanters, with arm positions along the body. Identical protocol settings for CT acquisition were used for all three phases. The volumetric CT dose index (CTDI_vol_) and the dose-length product (DLP) were recorded for all cadavers for the arterial phase.

**Table 1:** Acquisition and reconstruction parameter for the CT acquisitions with scan range covering the vertex to the trochanters

*Acquisition protocol*	
Tube potential (kV_p_)	120
Modulated tube current (mA)	200–400
Noise index	20
pitch	0.984
Rotation time (s)	0.8
*Reconstruction of the heart*	
Thickness (mm)	0.625
Slice increment (mm)	0.400
FOV (cm)	25
Matrix size (pixel × pixel)	512 × 512
ASiR level	0, 30, 50, 70, 100
Algorithm	Standard

Abbreviations: *FOV* field of view, *ASiR* adaptive statistical iterative reconstruction, *DFOV* detailed field of view

The preparation process was similar to previous studies [3, 4]. Specific single-use sets for femoral vascular cannulation were applied before the injection of a contrast agent mixture of an oil-based solution of defined viscosity (mixture of paraffin oil [paraffinum liquidum] with 6% oil-based contrast agent [Angiofil; Fumedica, Muri, Switzerland]).

According to the protocol of Grabherr et al., the injection included an arterial, venous, and dynamic phase with a volume of 1200 ml and a flow rate of 800 ml/min for the arterial phase [4].

### Image reconstruction

Using the raw image data of the arterial phase acquisition, a secondary reconstruction centered on the cardiac region was performed including the aortic arch or the carina down to the diaphragm with a mean scan length of 180.2 ± 24.1 mm (range 130–228 mm). In case of coronary artery bypass graft surgery, the *z*-axis was extended. We only analyzed the coronary reconstruction of the arterial phase.

Axial images were reconstructed for the heart with a slice thickness of 0.625 mm at a reconstruction increment of 0.400 mm with a FOV of 25 cm (matrix size 512 × 512 pixels) (see Table 1). Iterative reconstructions (IR) of the cardiac region were processed in five levels (0%; 30%; 50%; 70%; 100%) defining the strength of the IR algorithm increasing from 0 to 100%. Adaptive statistical iterative reconstruction (ASiR; GE HealthCare, Milwaukee, WI, USA) was used for all reconstructions. Reconstruction of all cardiac region datasets at all IR levels resulted in 135 image datasets (27 included cadavers × 5 ASiR levels).

### Image analysis

#### Objective image quality

**Fig. 2:**
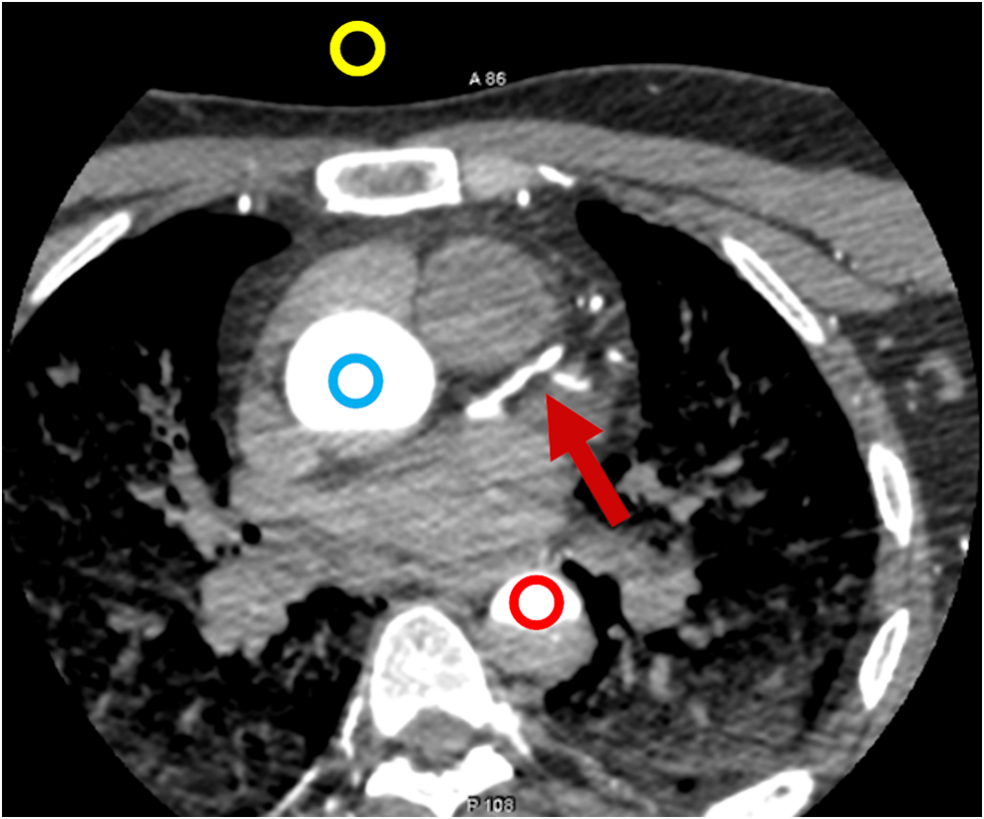
Position of the regions-of-interest placed in the air (yellow circle), aortic root (blue circle), and descending aorta (red circle). The red arrow points to the left anterior descending coronary artery (LAD). The paravertebral musculature is not completely visible on this axial slice

First, we evaluated the objective image quality (IQ) of the 135 datasets, adapting a previously validated method [21]. The measurements were obtained by one radiologist (AAA; 3 years of expertise in reading PMCT). For this purpose, circular regions-of-interest (ROIs) were drawn into the lumen of the coronary arteries (as large as possible, 2–20 mm^2^) (Fig. 2) in nine locations [21]: left main coronary artery (LM), proximal and distal (distal to the second diagonal branch) left anterior descending coronary artery (LAD), proximal first diagonal branch (D1), proximal and distal left circumflex coronary artery (LCX), first obtuse marginal branch (OM1), and proximal and distal (proximal to the origin of the posterior descending coronary artery) right coronary artery (RCA).

Furthermore, ROIs were drawn in the paravertebral musculature as background measurement (100 mm^2^), in the ascending and descending aorta (each 100 mm^2^) and in the adjacent air (100 mm^2^) to determine the changes in CT numbers for varying levels of IR. In addition, another circular ROI (100 mm^2^) was placed in the contrast-enhanced lumen of the aortic root to measure the image noise by determining the standard deviation of CT numbers [21]. Every ROI measurement had the exact same size and position in the five levels of iterative reconstruction by transferring the ROI into the different reconstruction levels.

The signal-to-noise ratio (SNR) was analyzed for all 9 coronary locations using the following formula:

1 $$ SNR=\frac{\mathrm{CT}\ \mathrm{number}\ \mathrm{of}\ \mathrm{coronary}\ \mathrm{lumen}\ \left[\mathrm{HU}\right]\ }{\mathrm{Standard}\ \mathrm{deviation}\ \mathrm{of}\ \mathrm{coronary}\ \mathrm{lumen}\ \mathrm{CT}\ \mathrm{measurement}\left[\mathrm{HU}\right]\ } $$

The contrast-to-noise ratio (CNR) was analyzed for all 9 coronary locations using the following formula:

2 $$ CNR=\frac{\mathrm{CT}\ \mathrm{number}\ \mathrm{of}\ \mathrm{coronary}\ \mathrm{lumen}\ \left[\mathrm{HU}\right]\hbox{--} \mathrm{CT}\ \mathrm{number}\ \mathrm{paravertebral}\ \mathrm{musculature}\ \left[\mathrm{HU}\right]}{\mathrm{image}\ \mathrm{noise}\ \mathrm{aortic}\ \mathrm{root}\ \left[\mathrm{HU}\right]} $$

#### Subjective image quality

The subjective image quality was independently evaluated by two radiologists (JB (J Boeven) and LC; 3 and 2 years of experience in reading, respectively). A four-point scale (1 = excellent image quality: “classic” image appearance, no or minimal artifacts, fully diagnostic; 2 = good image quality: slight artifacts, but fully diagnostic; 3 = moderate image quality: relevant artifacts, but evaluable concerning the presence of stenosis; 4 = unevaluable) based on the 18-segment model of the Society of Cardiovascular Computed Tomography was used (based on Society of Cardiovascular Computed Tomography [22]). The main criteria of subjective image quality were on the one hand “classic” image appearance (CT image appearance as known by and familiar to radiologists), which might be influenced by the use of too high IR levels causing blotchy image appearance and on the other hand artifacts due to image noise, resulting from low levels or absent IR. A comparable four-point scale was already applied in a previous study [21].

Subjective scores were given for seven localizations: the complete thorax, left main coronary artery (LM), left anterior descending coronary artery (LAD), left circumflex coronary artery (LXC), right coronary artery (RCA), and ascending and descending aorta, resulting in *n* = 27 cadavers x 5 reconstruction levels × 7 localizations x 2 readers = 1890 scores.

### Statistical analysis

A commercially available software (Microsoft Excel 2016, Redmond, WA, USA; SPSS, 27.0, Inc., Chicago, IL, USA) was used to perform statistical analysis. Continuous data are expressed as mean ± SD. Differences in objective and subjective image quality among the five levels of IR were calculated by means of a Bonferroni-corrected Friedman test with pairwise comparisons and a post hoc Dunn test. In addition, the number of diagnostic images (subjective image quality 1 and 2) between the five ASiR levels was compared and statistical differences tested by means of a Cochran Q test for dependent samples and a post hoc Dunn test. A two-tailed *p* value < 0.05 was considered statistically significant.

Inter-observer agreement was determined using a Fleiss kappa with chance correction according to Brennan and Prediger [23]. Inter-observer agreement was defined as excellent (κ > 0.81), good (κ = 0.61–0.80), moderate (κ = 0.41–0.60), fair (κ = 0.21–0.40), and poor (κ ≤ 0.20) [24].

## Results

### Radiation exposure

Averaged over all cadavers, the effective tube current time product of the arterial phase was 305.0 ± 38.2 mAs at 120kV_p_. The average CTDI_vol_ and DLP for the 27 cadavers were 21.7 ± 2.9 mGy and 2368.5 ± 519.5 mGy∙cm, respectively, for the arterial phase.

### Objective image quality

The CT numbers were comparable across the different IR levels in the aortic root, the ascending and descending aorta, paravertebral muscle and air (range aortic root, 555.7–555.8 HU; range aorta asc., 559.4–559.5 HU; range aorta desc., 551.0–551.5 HU; paravertebral muscle, 60.2–60.4 HU; air − 992.0–− 991.9 HU). Although the described ranges were within 1 HU when averaging over all 27 cadavers, there was a significant difference in the CT numbers between IR level 0 and 100 for the paravertebral muscle (*p* = 0.011), when employing the Friedman test. However, differences in CT numbers between the applied IR levels in the muscle were maximum 2 HU. Image noise decreased from IR level 0 to 100 in the aortic root (− 39%), paravertebral muscle (− 24%), and air (− 51%). The corresponding CT numbers in the coronary arteries (ROI size: 1–20 mm^2^) showed lower values with increasing IR levels with the highest mean value in level 0% (471.4 ± 120.8 HU, range 202.5 to 1060.3 HU) and the lowest mean value in level 100% (445.6 ± 133.2 HU, range 147.8 to 1072.5 HU). Compared to the highest CT number at IR level 0, CT numbers were 5.5% lower at IR level 100. Changes in mean CT number among the five IR levels were statistically significant (*p* < 0.001 for all comparisons).

**Fig. 3:**
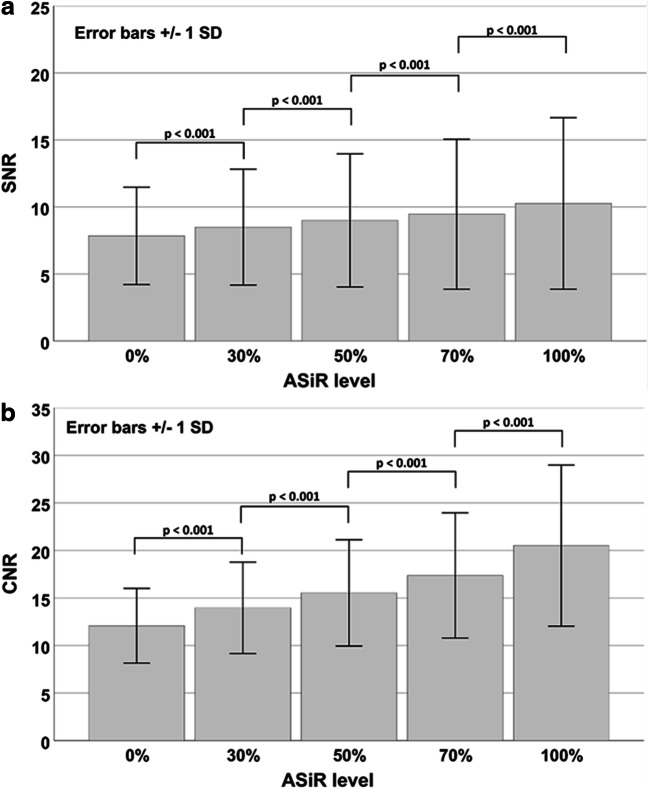
(a) Signal-to-noise ratio (SNR) and (b) contrast-to-noise ratio (CNR) calculated for the coronary arteries by means of formula 1 and 2, respectively. SNR and CNR increased significantly from increasing level to level

For the different IR levels, the mean SNRs for the nine coronary artery localizations were 7.8 ± 3.6 (0%), 8.5 ± 4.3 (30%), 9.0 ± 5.0 (50%), 9.5 ± 5.6 (70%), and 10.3 ± 6.4 (100%) (see Fig. 3a). The mean SNR significantly improved with each increasing level of IR, by 31% from IR level 0 to 100. The highest SNR was found at the highest level of IR (100%). For the different IR levels, the mean CNRs for the nine coronary artery localizations were 12.1 ± 3.9 (0%), 14.0 ± 4.8 (30%), 15.5 ± 5.6 (50%), 17.4 ± 6.6 (70%), and 20.5 ± 8.5 (100%). The mean CNR significantly improved with each increasing level of IR (see Fig. 3b), by 70% from IR level 0 to 100. The highest CNR was found at the highest level of IR (100%).

### Subjective image quality

An excellent image quality with a score of 1 was assessed 854 times (45.2%), score 2 (“good”) was assessed 611 times (32.2%), score 3 (“moderate”) 389 times (20.6%), and score 4 (“unevaluable”) 36 (1.9%) times.

**Fig. 4:**
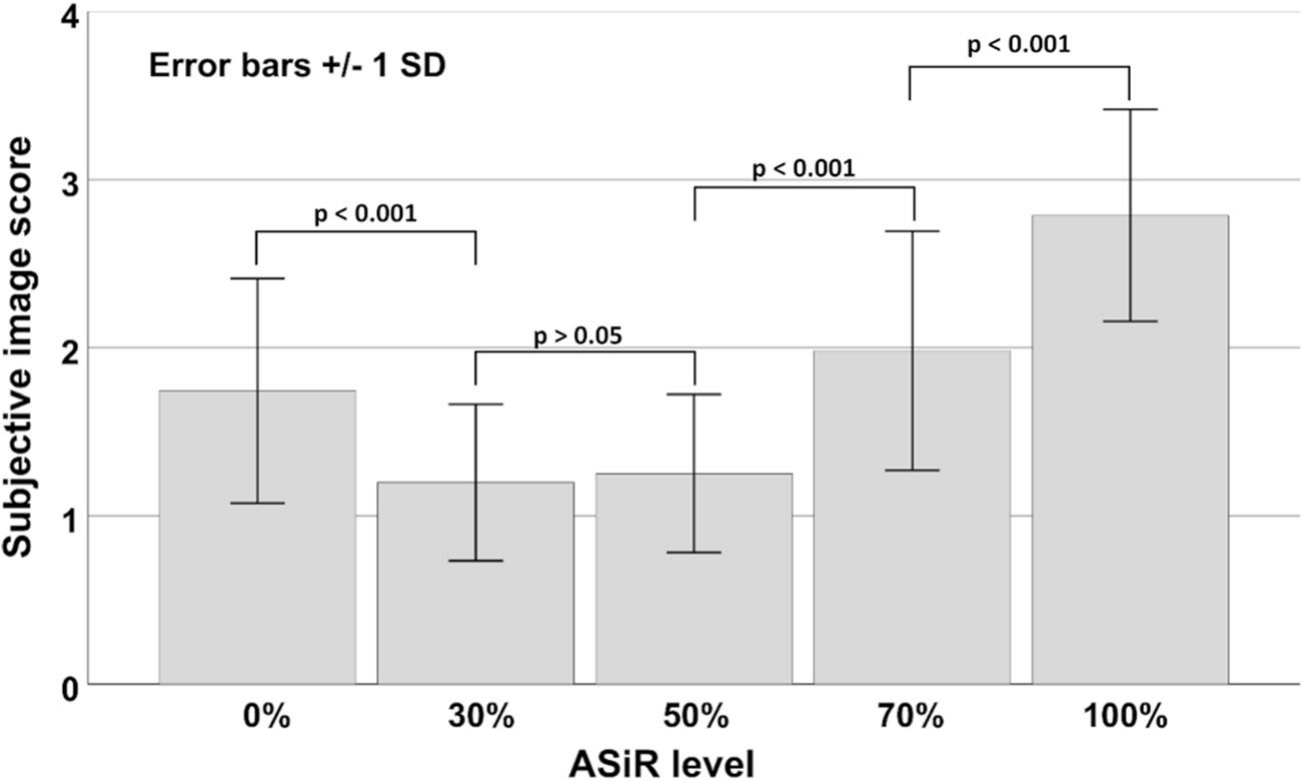
Averaged subjective image score for the seven evaluated ROIs. There was no significant difference in image score between ASiR level 30% and 50%

**Fig. 5:**
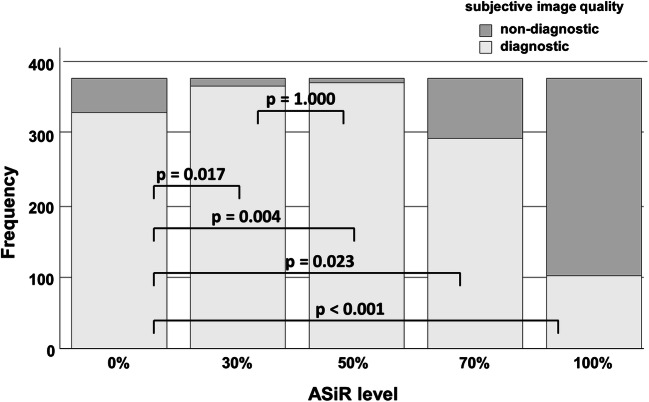
Comparison between subjective diagnostic image quality (score 1 and 2) and non-diagnostic image quality (score 3 and 4) for the five ASiR levels. There were significant differences between ASiR level 0% (filtered back projection) and all other ASiR levels

**Fig. 6:**
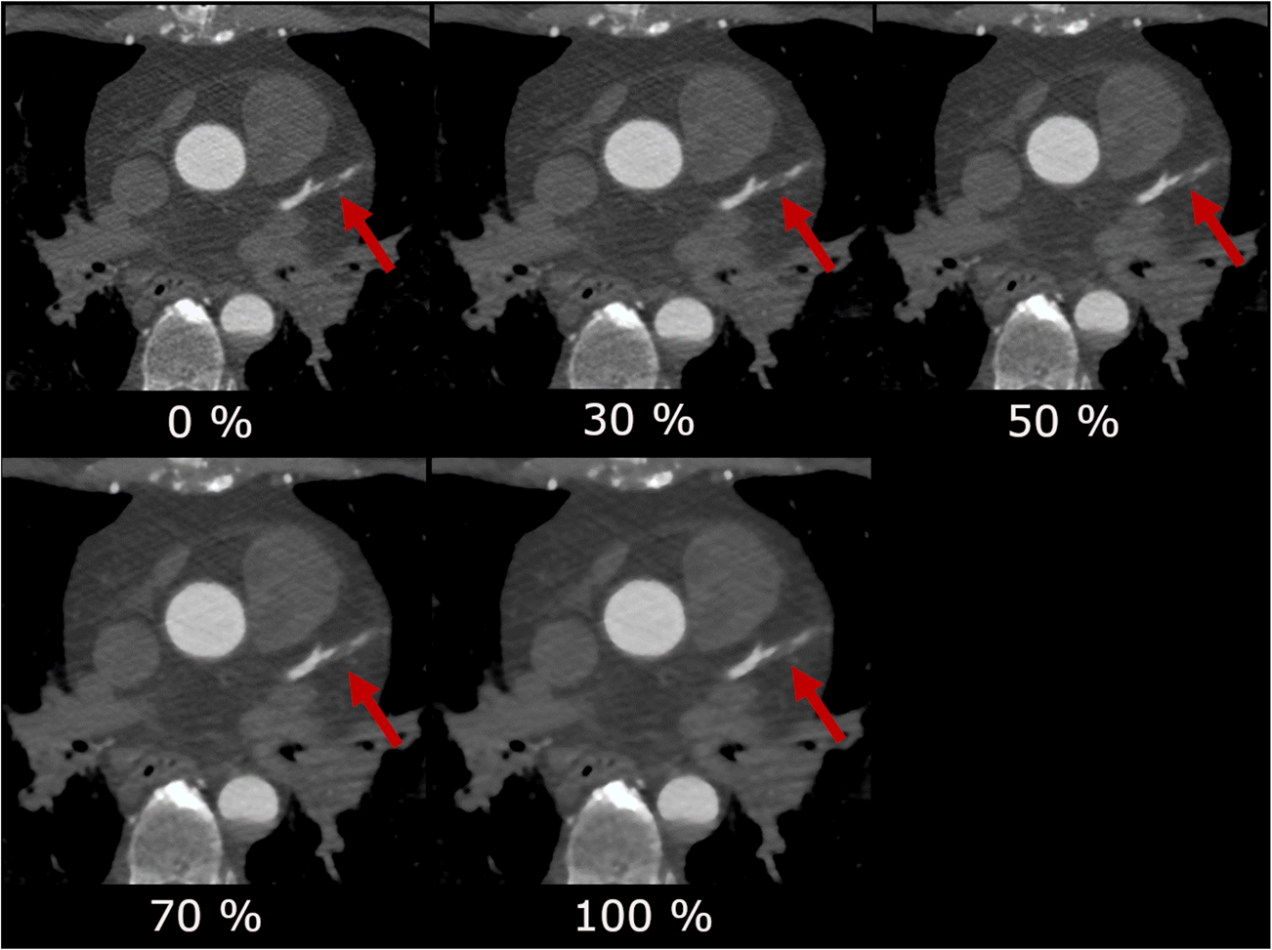
Influence of the ASiR level on the image noise. Axial slice through the heart. The arrow points to the left anterior descending coronary artery (LAD). With higher ASiR levels, image noise in the structures of the heart decreases; however, the image impression gets blotchier from level to level

**Fig. 7:**
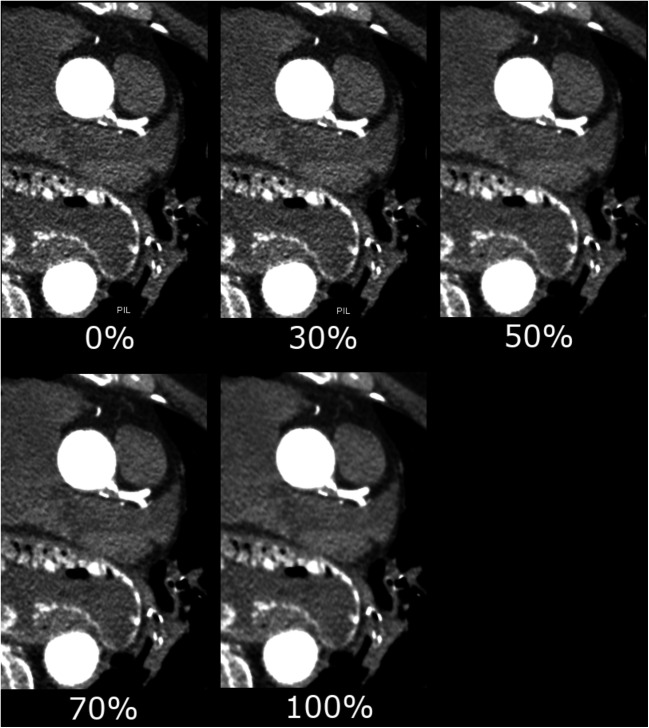
Influence of the ASiR level on the image noise. With higher ASiR levels, image noise in the structures of the heart decreases; however, the image impression gets blotchier from level to level. As additional finding, a hiatal hernia was diagnosed

Looking at the different IR levels, the subjective image quality was evaluated best at the IR level 30 with a mean score of 1.20 ± 0.47, following IR level 50 (1.25 ± 0.47), IR level 0 (1.74 ± 0.67), IR level 70 (1.98 ± 0.71), and at last IR level 100 (2.79 ± 0.63) (see Fig. 4). Subjective image score did not differ significantly between IR level 30 and 50. The score 4 (“unevaluable”) was given 32 times in IR level 100 and 4 times in IR level 70, whereas there was no localization in IR level 0, 30, or 50 that was evaluated with score 4. Subjective image quality was rated diagnostic (score 1 and 2) for IR level 50% in 98.4% of the evaluated ROIs, followed by IR level 30 (97.1%) and IR level 0 (87.3%). Cochran’s Q test indicated differences in the number of diagnostic and non-diagnostic ROI scores between the five IR levels (*Χ*^2^(4, *N* = 378) = 712.2, *p* < 0.001). Compared to IR level 0 (full FBP), IR levels 30 and 50 had significantly more diagnostic scores (*p* = 0.017 and *p* = 0.004, respectively), whereas IR levels 70 and 100 had significantly less diagnostic scores (*p* = 0.023 and *p* < 0.001, respectively) (see Fig. 5). No statistical significant difference was noted between IR level 30 and 50. The influence of the IR level on the image quality is depicted in Figs. 6 and 7. With increasing level, images appear less noisy. However, small details also appear blurred and the total image appearance looks plasticized, compared to the lower levels of IR.

The inter-observer agreement regarding the subjective image quality analysis was moderate (0.55 ± 0.16) and varied between 0.30 (IR level 70, fair agreement) and 0.73 (IR level 30 good agreement).

## Discussion

In the presented study, multiphase PMCTA was applied and evaluated in 27 cadavers resulting in a consistently diagnostic image quality and a good visualization of coronary arteries. Since the selection and volume of a dedicated contrast agent and the used CTA technique are relevant parts of image quality and image impression, we employed the most utilized and proven technique in PMCTA. Published clinical studies that analyzed the impact of advanced IR are often limited by employing diverse flow rates and amounts of contrast agents within one study [21]. In our study, we solely used the same amount of contrast media and the same flow rate.

Iterative reconstruction techniques are well established in CTA of patients due to the extensive and positive experience from clinical routine by means of radiation dose savings, noise reduction, and improvement of low-dose image quality. Regarding the image quality in cardiac CT, several studies pointed out an improvement of the CNR and subjective image quality by using IR techniques [25–27].

The employed CT protocol was a standardized protocol for PMCTA in the institute. In contrast to clinical protocols, the PMCT protocol did not specifically aim for a low radiation dose. This distinguishes the purpose of IR in clinical CT (aim for an additional dose reduction) and PMCT (aim for a classic image appearance as available in clinical CT). The dissimilar properties of alive patients and cadavers, e.g., heart movement, and the different requirements for an appropriate CT protocol impede the comparability of CT protocols and radiation exposure. Advanced IR techniques use multiple iterative reconstruction steps resulting in a progressive improvement of the CNR and subjective image quality in cardiac CT [28, 29]. A study by Kroepil et al. demonstrated that an increasing level of IR led to an improvement of the objective image quality, while the subjective image quality was limited by modification of the “classic” image appearance towards looking more plastic at higher IR levels [21]. Lack of familiarity of the image appearance might cause missing diagnoses, since the radiologist might misinterpret manifestations or structures, which appear different with different IR levels or vendors. The optimal IR level might differ between different types of CT examinations and should be evaluated when establishing a standard protocol [20]. In our study, objective image quality increased significantly with higher levels of IR due to the decreasing image noise (Fig. 3). The CT values in the coronary arteries decreased with increasing IR level, possibly due to partial volume effects with surrounding tissues with lower CT values. Subjective image analysis demonstrated that the coronary artery images appear increasingly blurry and plasticized at higher IR levels (Figs. 6 and 7). The overall image quality was rated best (1.2, excellent image quality) at the IR level of 30% (Fig. 4), with no significant differences between IR level of 30% and 50% for subjective image quality and number of diagnostic ROIs. Hence, our results are similar to what has been published for coronary CTA studies in living patients [21]. The authentic CT image appearance that radiologists are used to in clinical routine was preserved at low and medium levels of IR (ASiR 30% and 50%).

Forensic PMCTA reports should always be prepared in conjunction with a radiologist since otherwise, important findings can remain unreported [3]. Most forensic radiologists are adapted to common clinical CT image appearance produced by iterative reconstructions. Therefore, employing medium levels of IR is reasonable even in PMCT scans, as radiologists are familiar with the image appearance. Numerous forensic PMCT studies use filtered back projection (FBP) techniques or do not specify their CT protocols and image processing in detail [30–32].

Our study has some limitations. First, we used a retrospective study design with a limited cohort size. Second, a systematic comparison of coronary artery imaging and conventional autopsy has not been performed as the primary aim of this study was the impact on image quality and not the detection rate of coronary artery diseases. Third, the applied contrast agent was not visible in all regions for all cadavers due to cardiac pathologies, such as extravasation after cardiac damage and artifacts like contrast layering or filling defects [33]. There have been ten cadavers with only little or no contrast media in some ROIs, causing highly deviating CT values for these cadavers in the assessed ROIs. For this purpose, we excluded those ten cadavers. Fourth, we only investigated one IR system (ASiR) and one CT scanner of only one manufacturer. Although we cannot generalize our results to reflect all IR algorithms (especially newer ones), the general principle of decreasing image noise with increasing levels of IR also applies to other vendors [21, 34, 35]. Fifth, the rated subjective image quality always depends on the experience of the rating radiologist and the image quality a radiologist is used to. The two radiologists’ inter-reader agreement in this study varied between fair and good. Possible reasons could be the two radiologists’ relatively young reading experience (2 and 3 years), which might reinforce differences in image quality evaluation. Still, both radiologists are familiar in reading postmortem CTs. For different radiologists, e.g., more experienced ones, subjective image quality might differ from the results presented here. When defining institutional protocol settings, radiologists of the institution might rate image quality in consensus to obtain the optimal settings. Last, we did not include the body mass index (BMI) of the cadavers into our analysis, due to incomplete data. For a group of thinner or bigger patients, image quality might differ from the included cohort. However, we do not expect large differences since we had both normal (BMI 19.8 kg/m^2^) and obese patients (BMI 38.9 kg/m^2^) included.

Until now, clinical studies have usually evaluated to what degree IR methods enable reducing the effective dose to the patient. This is not the aim of IR in forensic radiology, since the radiation exposure has no negative implication to the examined cadavers. The question remains whether FBP with high-dose levels work better or equal than IR with lower-dose levels. As mentioned in the introduction, several published studies employ FBP as method of image reconstruction, since IR methods are not available in all forensic institutes (cost-intensive purchase, requirement of a state-of-the-art CT scanner). In case IR is available on the CT scanners, then it is presumably not the most modern IR algorithm. In a future study, it is of interest to assess whether increasing the dose in PMCT in combination with FBP outperforms the current protocol with optimal IR level, in terms of diagnostic accuracy and confidence and image appearance.

## Conclusion

In PMCTA, objective image quality progressively improves with increasing level of ASiR in the coronary arteries with the best CNR at the highest noise suppression level. Subjective image quality was rated significantly better at low and medium levels of IR, as the “classic” image appearance was preserved. Thus, for PMCTA, a medium level of ASiR seems optimal.

## Declarations

Written informed consent was not available due to the imaging of human corpses. As this was a

retrospective study and data were treated anonymously, a specific ethical approval was not necessary

according to an agreement with the local justice department and the ethical standards.
